# CT-IGFBP-4 as a Predictive Novel Biomarker of Ischemic Cardiovascular Events and Mortality: A Systematic Review

**DOI:** 10.1155/2022/1816504

**Published:** 2022-08-21

**Authors:** Abhinav Bhattarai, Pritam Singh Sunar, Sangam Shah, Rajan Chamlagain, Nishan Babu Pokhrel, Pitambar Khanal, Sanjit Kumar Sah, Sujan Poudel, Kapil Belbase, Swati Chand, Rajaram Khanal, Anil Bhattarai

**Affiliations:** ^1^Maharajgunj Medical Campus, Institute of Medicine, Tribhuvan University, Maharajgunj, Kathmandu 44600, Nepal; ^2^Tribhuvan University Teaching Hospital, Maharajgunj, Kathmandu 44600, Nepal; ^3^Division of Research Affairs, Larkins Community Hospital, South Miami, USA; ^4^Rochester General Hospital, Rochester, NY, USA; ^5^Department of Cardiology, Maharajgunj Medical Campus, Institute of Medicine, Tribhuvan University, Maharajgunj, Kathmandu 44600, Nepal

## Abstract

**Methods:**

The electronic databases PubMed, medRxiv, ScienceDirect, and Google Scholar were searched for relevant literature from inception to the 10^th^ of December, 2021. Thus, retrieved literature was screened by title and abstract, followed by full-text screening based on the eligibility criteria. The risk of bias was accessed using the quality in prognostic studies (QUIPSs) tool. The data on cardiovascular outcomes about CT-IGFBP-4 levels were studied and the results were synthesized.

**Results:**

Five studies with a total of 1,417 participants were included in our study. The studies reported a low risk of bias. The mean age of the participants was 66.14 and more than 65% were males. Elevated CT-IGFBP-4 levels were associated with poor cardiovascular outcomes and increased mortality in severely ill patients. In contrast, there were no significant findings in the case of stable patients. Sandwich ELISA using lithium-heparin plasma provided a better detection limit of 0.15 ng/ml, low cross-reactivity (<2%), and generated linear results between 12 and 500 ng/ml.

**Conclusion:**

CT-IGFBP-4 is an efficient biomarker for the prediction of MACE and mortality in patients with severe ischemic cardiovascular events.

## 1. Background

Insulin-like growth factor (IGF), is an important peptide responsible for various cellular growth, proliferation, and differentiation upon interaction with IGF receptors on the cell surface. In the blood, IGF remains bound to one of the six IGF binding proteins (IGFBPs 1–6), which regulates the IGF activity [1]. IGFBP-4 has been previously known to have a significant role in cancer physiology [2] and is used as a diagnostic and prognostic marker in lung cancer [3, 4] and various autoimmune diseases like chronic lupus nephritis [5]. The role of IGFBP-4 in cardiovascular events has been recently identified [6].

**Figure 1:**
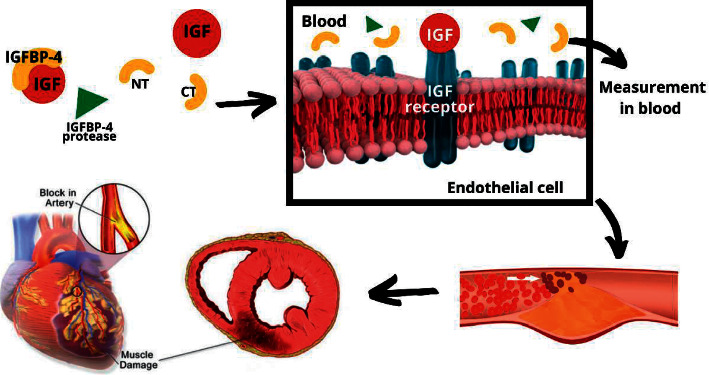
Cleavage of IGFBP-4 liberates free IGF which causes endothelial proliferation, promotes atherosclerosis, and ischemia eventually leading to myocardial infarction.

IGF-1, interacting with its receptors, promotes vascular proliferation, macrophage chemotaxis, excess low-density lipoprotein (LDL) uptake, and the release of proinflammatory cytokines in the endothelium, which can cause ischemia and myocardial infarcts. To circumvent this, IGFBP-4 is generated by the vascular endothelial cells, which bind to IGF, blocking its interaction with the receptor [7]. Secretion of IGFBP-4 proteases by atherosclerotic plaques cleaves the binding protein into two fragments: one belonging to the carboxyl-terminal (CT) and another to the amino-terminal (NT) releasing free IGF which can cause inflammatory and proliferative activity in the endothelium, ischemia, and eventually cardiovascular complications [8].  Figure 1 summarizes the process of free IGF-mediated vascular proliferation and consequent outcomes.

Elevation in the level of these ‘cleaved' fragments in the blood indicates increased IGFBP-4 proteolysis and elevated IGF activity, providing a valuable biomarker role in the early diagnosis, prognosis, and prediction of severe ischemic cardiovascular events [9]. The CT fragment of the insulin-like growth factor binding protein-4 (IGFBP-4). The CT-IGFBP-4 is a novel molecule that has shown promising efficacy in the early diagnosis of ischemic cardiovascular events in recent studies [10–14], which we have emphasized here. The objective of this systematic review is to assess the biomarker utility of CT-IGFBP-4 in predicting ischemic cardiovascular events and mortalities.

## 2. Methods

### 2.1. Search Strategy

The databases PubMed, medRxiv, ScienceDirect, and Google Scholar were searched for relevant literature published from inception to December 10^th^, 2021. We searched the databases using the terms “CT-IGFBP-4,” “IGFBP-4,” “coronary artery disease,” “coronary,” “cardiac,” “heart,” “failure,” “myocardia,” “ischemia,” “infarction,” and “mortality” in the title, keywords, and abstract. The search terms were connected by ‘AND' and ‘OR' Boolean operators.

### 2.2. Study Selection

The systematic review is registered in PROSPERO (CRD42022311531). The preferred reporting items for systematic reviews and meta-analyses (PRISMA) [15] guideline was followed for the study selection process. The retrieved articles were exported to Mendeley Desktop version 1.19.8. Duplicates were removed both automatically and manually. Two independent reviewers (AB and PSS) screened the articles by title, and abstract followed by full-text screening based on the eligibility criteria. The discrepancies during the study selection process were solved through consensus from the third reviewer (SS).

### 2.3. Eligibility Criteria

Studies that were written in English were considered eligible for this review if they fulfilled the following inclusion criteria:

1. The study population included patients diagnosed or suspected of having heart disease at the time of admission.
2. CT-IGFBP-4 assay was performed as a measure of prognostic factor.
3. Statistical analysis for the predictive ability of CT-IGFBP-4 was performed.

Case reports, editorials, case series, and review articles were excluded from this review.

### 2.4. Data Extraction

The data from the selected studies were extracted based on a prespecified data extraction form which included the following data variables: [1] author and year of study, [2] study design, [3] study population, [4] population size, [5] age, [6] gender, [7] study findings, [8] predictively measure of CT-IGFBP-4 in cardiovascular mortality, and [9] assay details of CT-IGFBP-4 measurement.

For the findings, we extracted data on levels of CT-IGFBP-4 in relation to the cardiovascular outcomes of the patients including major adverse cardiovascular events (MACEs), rehospitalization, revascularization, and deaths. Similarly, for predictability, the receiver operating characteristic (ROC) analysis performed by the studies was accessed, and area under curve (AUC) values along with respective 95% confidence intervals (CIs) were extracted. We judged AUC values between 0.5-0.6 for the diagnostic test to interpret no discrimination; values between 0.7-0.8 as acceptable and values between 0.8-0.9 as excellent performance of the diagnostic testing [16].

### 2.5. Risk of Bias Assessment

Two reviewers (PSS and SS) who were blinded to the study author, performed the assessment of risk of bias among the studies using the quality in prognostic studies (QUIPSs) tool [17] in 6 bias domains as follows: study participation, study attrition, prognostic factor measurement, outcome measurement, study of confounding factors, and statistical analysis and reporting. Low, moderate, and high risks, were assigned to each domain for each study, and overall risk was evaluated. We judged the studies to have a low risk of bias when all the bias domains possessed a low risk and moderate and high risk, respectively, if any one bias domain had a moderate risk and at least two bias domains had a high risk, respectively. The overall risk among the studies was judged to be low if more than half of the studies possessed a low risk, moderate if at least half of the studies possessed a moderate risk, and high if any two studies possessed a high risk of bias.

### 2.6. Data Synthesis

From the included studies, the data on the level of CT-IGFBP-4 in patients with and without poor outcomes were taken and included in the descriptive summary. The data were summarized using descriptive statistics including means and percentages. The median and interquartile ranges were used to express continuous variables.

## 3. Results

### 3.1. Search Results and Study Selection

**Figure 2:**
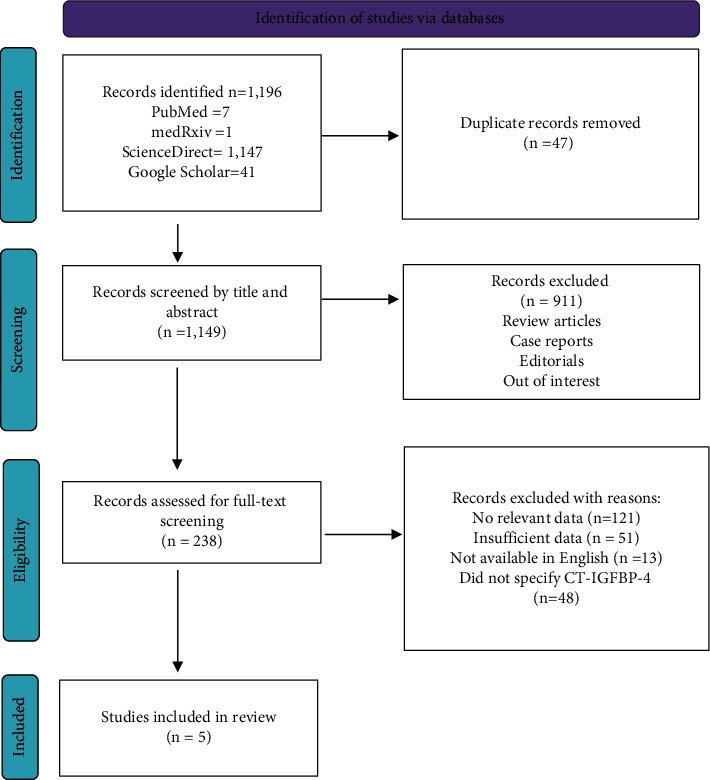
Preferred reporting items for systematic reviews and meta-analyses (PRISMA) flowchart for study selection.

The initial database search retrieved a total of 1,196 articles. The detailed study selection process is displayed in Figure 2. Briefly, 238 of the retrieved articles were subjected to full-text screening after the screening of the title and abstract. Five of the studies met our inclusion criteria and were included in this review [10–14, 18].

### 3.2. The Risk of Bias within the Studies

Four studies [10–13] reported a low risk of bias, and one study [10] reported a moderate risk of bias. The overall risk of bias among the studies was low. The results of the risk of bias assessment of individual studies in all the six domains are shown in Table 1.

### 3.3. Descriptive Characteristics of the Included Studies and the Participants

The descriptive characteristics of the included studies and the participants are shown in Table 2. All the studies performed a prospective cohort analysis of patients with a follow-up duration ranging from 6 months to five years. A total of 1,417 participants are included in our systematic review. The mean age of the patients was 66.14, and 65%% of the participants were male. The study population varied but was related to ischemic cardiovascular events. In all included studies, CT-IGFBP-4 levels were compared between participants who had cardiovascular complications and those who did not. The predictability of the biomarker and assay utilized for estimation was clarified by all included studies.

### 3.4. CT-IGFBP-4 Levels in Ischemic Cardiovascular Events

Three studies [12–14] reported outcomes on the basis of the occurrence of MACE among the patients. Median CT-IGFBP-4 levels in patients developing MACE ranged from 62 to 160 ng/mL while levels between 45 and 84 ng/mL were obtained in patients who did not develop MACE. A study by Schulz et al. [10] reported an increased incidence of revascularization, rehospitalization, and mortality in patients whose CT-IGFBP-4 level was above 31.55 ng/mL. However, the statistics were not significant (*p* > 0.05), and this is the only included study that did not support the utility of CT-IGFBP-4 in predicting cardiovascular mortality. In a prospective study by Konev et al. [11], patients who died during the follow-up had reported median CT-IGFBP-4 levels ranging from 104 to 203 ng/mL at the time of admission while the survivors had comparatively less (47–133 ng/mL) level of CT-IGFBP-4 at admission. In the same study, the hazard ratio was 6.15 for one-month mortality and 4.20 for one-year mortality in patients with CT-IGFBP-4 levels greater than 92.5 ng/mL during admission. Likewise, in the study by Hjortebjerg et al. [13], which was performed on STEMI patients undergoing percutaneous coronary intervention (PCI), deceased patients had a higher level of CT-IGFBP-4 at admission (69 (46–108) ng/ml) as compared to survivors (45 (31–66) ng/ml). Depending upon the study population, the cut-offs varied. In brief, CT-IGFBP-4 levels were observed to be significantly elevated in patients who developed poorer outcomes which are shown in Table 2.

### 3.5. Prediction of Mortality

All studies accessed the predictability of CT-IGFBP-4 in respective events by area under curve (AUC) values from the receiver operating characteristic (ROC) curve, which is shown in Table 2. Each AUC value is applied to the corresponding category of patients. The highest AUC was reported by Postnikov et al. [12] for CT-IGFBP-4 in predicting MACE in patients with symptoms of myocardial ischemia (AUC 0.809). In brief, excellent predictability was reported in predicting MACE in patients with symptoms of myocardial infarction and in predicting cardiovascular mortality in STEMI patients undergoing PCI.

### 3.6. Assays for IGFBP-4

CT-IGFBP-4 levels were estimated using ELISA in four studies [10–12, 14] except for Hjortebjerg et al. [13], which was based on immunofluorescence. A sandwich ELISA was used which utilized two monoclonal antibodies: insulin binding protein (IBP) 163 as the capture antibody, and IBP 182 as the detection antibody. The same antibodies were used in the study that utilized immunofluorescence. EDTA obtained and lithium-heparin obtained plasma were used for the assays. The samples were stored at low temperatures (−40°C to −80°C) until testing. A study by Postnikov et al. [12] reported that the assay produced linear results for CT-IGFBP-4 concentration between 12 and 500 ng/ml. Konev et al. and Postnikov et al. [11, 12] reported the cross-reactivity of the assay which was less than 2%. The detection limit ranged from 0.8 ng/ml to 0.15 ng/ml. The description of assays utilized in the measurement of CT-IGFBP-4 is shown in Table 3.

## 4. Discussion

Our findings were consistent with the encouraging results for the use of CT-IGFBP-4 in the prognosis of ischemic cardiovascular events and prediction of mortality. CT-IGFBP-4 levels in patients who died during follow-up were high at admission as compared to patients who survived. However, the results were contrary to the study by Schulz et al. [10]. This is possible because the study included stable and healthier patients, while other studies had severely ill participants. Thus, the extent of elevation of the biomarker differed according to the severity of the disease. Importantly, there were vast differences in the cut-offs of CT-IGFBP-4, and this can be explained by the difference in the cardiovascular status of patients. For example, Konev et al. [11] included patients with acute heart failure while Schulz et al. [10] included patients with stable cardiovascular disease, and had higher and lower cut-off, respectively. Likewise, Postnikov et al. [12] excluded STEMI patients while Hjortebjerg et al. [13] included them.

Out of the total participants, patients with higher CT-IGFBP-4 developed MACE during follow-up. Moreover, elevated levels were associated with an increased incidence of hospitalizations and even revascularization. Pregnancy associated plasma protein -A (PAPP-A) is considered a highly-efficient protease of IGFBP-4, and the level of CT-IGFBP-4 may relate to PAPP-A level and its activity [18, 19]. Also, it was proposed that heparin and its products could significantly increase the levels of PAPP-A and eventually CT-IGFBP-4 [20, 21]. However, Hjortebjerg et al. [22] showed that the marked increase in PAPP-A in heparin administration did not increase CT-IGFBP-4, furthermore, encouraging its potentiality as a superior risk marker in acute coronary syndrome (ACS).

The highest AUC of 0.809 (95% CI 0.726–0.892) was reported in predicting MACE in patients with symptoms of myocardial ischemia, which was comparable with the predictability of other established biomarkers such as brain natriuretic peptide (BNP), NT-proBNP, highly sensitive cardiac troponin I (hs-cTI), CK-MB, myoglobin, and ischemia modified albumin (IMA) in related cardiac risk assessment. The AUC value of BNP and NT-proBNP in predicting ischemic heart failure was 0.770 and 0.754, respectively [23–25]. The AUC value was 0.87–0.95 in the case of hs-cTI in suspected acute myocardial infarction [26, 27]. CK-MB had an AUC of 0.84, and myoglobin had an AUC of 0.79 in the early diagnosis of myocardial infarction in patients with chest pain [28]. Likewise, the AUC was 0.754 in the context of IMA in the early diagnosis of acute coronary syndrome [29].

The feasibility of the routine investigation of CT-IGFBP-4 can be considered by the application of ELISA, an easy-to-perform assay that yields high sensitivity and specificity. The ELISA using monoclonal antibodies IBP163 and IBP182 could detect all CT-IGFBP-4 present in the blood and was not influenced by the metabolic states and glycosylated forms [30]. Konev et al. showed that the PAPP-A derived CT fragment of IGFBP-4 does not undergo truncation, complex formation, or any modifications, thus validating the clinical use of this biomarker [9]. The sensitivity and detection limit of Sandwich ELISA was, however, dependent upon the type of specimen selected and the storage temperature. The selection of lithium-heparin obtained plasma and its storage at −80°C generated a much better detection limit of 0.15 ng/ml [11] as compared to EDTA obtained plasma, which was stored at −70°C, and had a detection limit of 0.8 ng/ml [12]. With EDTA obtained plasma, TR-IFMA produced a better detection limit of 0.4 ng/ml [13]. TR-IFMA could produce a better detection limit when lithium-heparin obtained plasma is used and stored at low temperature.

The main strength of our study is that it is the first systematic review emphasizing the utility of CT-IGFBP-4 as a marker of cardiac risk assessment. This study compares the clinical outcomes between patients with a higher and a lower level of CT-IGFBP-4 in different cardiovascular conditions. Also, we have exhibited the feasibility and techniques of biomarker estimation from a routine diagnostic point of view. On the other hand, our study has several limitations. One limitation is that the total number of participants was lower. The participants were heterogeneous and did not have the same clinical condition. Also, the assays used for estimation, sample type, and storage were different between the studies. We could not perform a robust meta-analysis since the study population was not specifically the same and pooling the data could produce erroneous interpretations. Moreover, insufficiency in the study might exist due to the loss of valuable data from the potential omission of studies.

## 5. Conclusion

CT-IGFBP-4 is an efficient biomarker for the prediction of MACE and mortality in patients with severe ischemic cardiovascular events. Measurement of CT-IGFBP-4 by ELISA using lithium-heparin obtained plasma generated better detection and results. Incorporation of the biomarker within routine cardiac risk assessment of severely ill patients may prove beneficial in predicting their prognosis. However, more studies and analyses comparing the levels of these fragments in specific cardiovascular diseases are needed to validate their clinical utility.

## Figures and Tables

**Table 1:** QUIPS tool for assessment of the risk of bias in the studies.

Study	García-osuna [14]	Schulz et al. [10]	Konev et al. [11]	Postnikov et al. [12]	Hjortebjerg et al. [13]
Study participation	Low	Low	Low	Low	Low
Study attrition	Moderate	Low	Low	Low	Low
Prognostic factor measurement	Low	Low	Low	Low	Low
Outcome measurement	Low	Low	Low	Low	Low
Study of confounding factors	High	Low	Low	Low	Low
Statistical analysis and reporting	Low	Low	Low	Low	Low
The overall risk of bias	Moderate	Low	Low	Low	Low

**Table 2:** Descriptive characteristics and findings in the included studies.

S.N.	Study	Study population	Population size, mean age	Gender	Findings			Predictability
1.	García-osuna [14]	Hospitalized patients with ST-elevation myocardial ischemia	196, 65	Male: 74% Female: 26%	Total (*n*) = 196			AUC 0.63 (95% CI 0.49–0.76) in MACE
					MACE (*n*) = 26	Non-MACE (*n*) = 170		
					CT-IGFBP-4 ≥ 62 ng/mL Hazard ratio (HR) = 2.95	CT-IGFBP-4 ≤ 62 ng/mL		
					Conclusion: CT-IGFBP-4 is a strong predictor of MACE (*p*=0.043)			

2.	Schulz et al. [10]	Patients with stable cardiovascular disease	229, 64	Male: 61% Female: 39%	Total (*n*) = 229			0.61 (95% CI 0.54–0.69) in CAD
					Events	CT-IGFBP-4	CT-IGFBP-4	
						≥31.55 ng/mL	≤31.55 ng/mL	
					Death	7	5	
					Re-hospitalization	23	13	
					Re-vascularization	26	20	
					Conclusion: CT-IGFBP-4 is not significantly associated with an increased risk of rehospitalization, revascularization, and death in patients with stable cardiovascular disease. (*p* > 0.05)			

3.	Konev et al. [11]	Patients hospitalized with acute heart failure (AHF)	156, 76.7	NR	Total (*n*) = 156			0.753 (95% CI 0.657–0.850) in cardiovascular mortality
						Non-survivors (*n*) = 52	Survivors (*n*) = 104	
					CT-IGFBP-4 at admission Median(IQR)	136 (104–203)	88 (47–133)	
					Hazard ratio (HR)			
					A cut-off value of CT-IGFBP-4 at admission	One-month mortality	One-year mortality	
					≥92.5 ng/mL	6.15	4.20	
					Conclusion: CT-IGFBP-4 is significantly associated with increased mortality in AHF. (*p*=0.0018)			

4.	Postnikov et al. [12]	Patients in ER with symptoms of myocardial ischemia	180, 63	Male: 53% Female: 47%	Total patients (*n*) = 180			0.809 (95% CI 0.726–0.892) in MACE
					Patients with MACE (*n*) = 16	Patients without MACE (*n*) = 164		
					CT-IGFBP-4 (ng/mL) Median(IQR) = 160 (103–258)	CT-IGFBP-4 (ng/mL) Median(IQR) = 84 (61–122)		
					Conclusion: CT-IGFBP-4 is significantly associated with increased risk of MACE in nonSTEMI patients. (*p* < 0.0001)			

5.	Hjortsberg et al. [13]	STEMI patients being treated with percutaneous coronary	656, 62	Male: 73% Female: 27%	Total (*n*) = 656			0.80 (95% CI 0.75–0.86) in cardiovascular mortality
					MACE (*n*) = 166	Without-MACE (*n*) = 490		
					CT-IGFBP-4 (ng/mL) Median(IQR) = 69 (46–108) Hazard ratio (HR) = 2.07	CT-IGFBP-4 (ng/mL) Median(IQR) = 45 (31–66)		
					Conclusion: CT-IGFBP-4 is significantly associated with increased risk of mortality in STEMI patients. (*p* < 0.001)			

Age is expressed in mean and gender in percentage. IQR, interquartile range. NR, not reported. MACE, major adverse cardiovascular events. AHF, acute heart failure. STEMI, ST-elevation myocardial ischemia. CT-IGFBP-4, carboxyl-terminal insulin-like growth factor-binding protein-4.

**Table 3:** Description of the assay used in CT-IGFBP-4 measurements in the included studies.

S.N.	Author	Assay used for ct-igfbp-4 determination	Detection limit	Sample used	Storage
1.	García-osuna [14]	ELISA (type not specified)	NR	EDTA-obtained plasma	−80°C

2.	Schulz et al. [10]	Sandwich ELISA	0.8 ng/ml	Lithium-heparin obtained plasma	−40°C

3.	Konev et al. [11]	Sandwich ELISA using monoclonal antibodies (IBP163 as capture antibody, and IBP182HRP as detection antibody)	0.15 ng/ml	Lithium-heparin obtained plasma	−80°C
		Cross reactivity: <2%			

4.	Postnikov et al. [12]	Sandwich ELISA using monoclonal antibodies (IBP163 as capture antibody, and IBP182 as detection antibody)	0.8 ng/ml	EDTA obtained plasma	−70°C
		Cross-reactivity: <1.5%			
		Linearity range: 12–500 ng/mL			

5.	Hjortebjerg et al. [13]	Time-resolved-immunoflourimetric assay (TR-IFMA) using monoclonal antibodies IBP163 and IBP182	0.4 ng/ml	EDTA obtained plasma	NR

ELISA, enzyme-linked immuno-sorbent assay. EDTA, ethylene diamine tetra-acetic acid. IBP, insulin binding protein. NR, not reported.

## Data Availability

All the required information is in the manuscript itself.
